# Use of Xpert MTB/RIF in Decentralized Public Health Settings and Its Effect on Pulmonary TB and DR-TB Case Finding in India

**DOI:** 10.1371/journal.pone.0126065

**Published:** 2015-05-21

**Authors:** Kuldeep Singh Sachdeva, Neeraj Raizada, Achuthan Sreenivas, Anna H. van't Hoog, Susan van den Hof, Puneet K. Dewan, Rahul Thakur, R. S. Gupta, Shubhangi Kulsange, Bhavin Vadera, Ameet Babre, Christen Gray, Malik Parmar, Mayank Ghedia, Ranjani Ramachandran, Umesh Alavadi, Nimalan Arinaminpathy, Claudia Denkinger, Catharina Boehme, C. N. Paramasivan

**Affiliations:** 1 Central TB Division, Government of India, New Delhi, India; 2 Foundation for Innovative New Diagnostics, Geneva, Switzerland; 3 World Health Organization, India Country Office, New Delhi, India; 4 Department of Global Health, Academic Medical Center and Amsterdam Institute of Global Health and Development, Amsterdam, The Netherlands; 5 KNCV Tuberculosis Foundation, The Hague, The Netherlands; 6 Department of Infectious Disease Epidemiology, School of Public Health, Imperial College, London, United Kingdom; California Department of Public Health, UNITED STATES

## Abstract

**Background:**

Xpert MTB/RIF, the first automated molecular test for tuberculosis, is transforming the diagnostic landscape in high-burden settings. This study assessed the impact of up-front Xpert MTB/RIF testing on detection of pulmonary tuberculosis (PTB) and rifampicin-resistant PTB (DR-TB) cases in India.

**Methods:**

This demonstration study was implemented in 18 sub-district level TB programme units (TUs) in India in diverse geographic and demographic settings covering a population of 8.8 million. A baseline phase in 14 TUs captured programmatic baseline data, and an intervention phase in 18 TUs had Xpert MTB/RIF offered to all presumptive TB patients. We estimated changes in detection of TB and DR-TB, the former using binomial regression models to adjust for clustering and covariates.

**Results:**

In the 14 study TUs, which participated in both phases, 10,675 and 70,556 presumptive TB patients were enrolled in the baseline and intervention phase, respectively, and 1,532 (14.4%) and 14,299 (20.3%) bacteriologically confirmed PTB cases were detected. The implementation of Xpert MTB/RIF was associated with increases in both notification rates of bacteriologically confirmed TB cases (adjusted incidence rate ratio [aIRR] 1.39; CI 1.18-1.64), and proportion of bacteriological confirmed TB cases among presumptive TB cases (adjusted risk ratio (aRR) 1.33; CI 1.6-1.52). Compared with the baseline strategy of selective drug-susceptibility testing only for PTB cases at high risk of drug-resistant TB, Xpert MTB/RIF implementation increased rifampicin resistant TB case detection by over fivefold. Among, 2765 rifampicin resistance cases detected, 1055 were retested with conventional drug susceptibility testing (DST). Positive predictive value (PPV) of rifampicin resistance detected by Xpert MTB/RIF was 94.7% (CI 91.3-98.1), in comparison to conventional DST.

**Conclusion:**

Introduction of Xpert MTB/RIF as initial diagnostic test for TB in public health facilities significantly increased case-notification rates of all bacteriologically confirmed TB by 39% and rifampicin-resistant TB case notification by fivefold.

## Introduction

Tuberculosis (TB) remains a major global public health problem even today. Despite longstanding availability of treatment, an estimated 8.6 million TB cases and 1.3 million TB deaths were reported in 2012 [1]. Early and improved case detection of TB including multi-drug-resistant TB (MDR-TB) has therefore become one of the global priorities for TB control. In December 2010, the World Health Organization (WHO) endorsed the Xpert MTB/RIF (Cepheid, Sunnyvale, CA, USA) [2] assay, which has demonstrated high sensitivity and specificity for both detection of pulmonary TB and rifampicin resistance [3]. In 2013, WHO released revised policy guidelines on the use of Xpert MTB/RIF in adults and children. These guidelines recommend that, Xpert MTB/RIF may be used rather than conventional microscopy and culture as the initial diagnostic test in all adults presumed to have TB (conditional recommendation acknowledging resource implications, high-quality evidence) [2–4]. The WHO also provided guidance for implementation of Xpert MTB/RIF in high-burden settings and recommended country specific operational research related to the introduction of Xpert MTB/RIF, its impact in the diagnosis of TB, MDR-TB and patient management [5].

With an estimated 26% of global TB cases, India is the highest TB burden country globally [1]. India's Revised National TB Control Programme (RNTCP) currently recommends that any person suspected of having pulmonary tuberculosis be initially examined with sputum smear microscopy; if the patient is smear-negative, subsequent diagnosis is based on repeated microscopy, radiology, and clinical judgment. If a high-sensitivity rapid TB diagnostic test, such as Xpert, replaces smear-microscopy it offers the possibility of early detection of more TB patients, while simultaneously detecting rifampicin resistance. This paper captures our experience of rolling out Xpert MTB/RIF assay at 18 sites across India at the district and sub-district level of the health system for the purpose of diagnosing TB and rifampicin resistance among pulmonary TB cases. The primary objectives of the study was to assess the effect of Xpert MTB/RIF, as a substitution for smear microscopy, on the detection and notification of all cases, bacteriologically confirmed cases and rifampicin-resistant pulmonary TB cases. A secondary, objective was to evaluate the positive predictive value of detection of rifampicin resistance by Xpert MTB/RIF in the patient population routinely served by the RNTCP.

## Methods

### Setting

**Fig 1:**
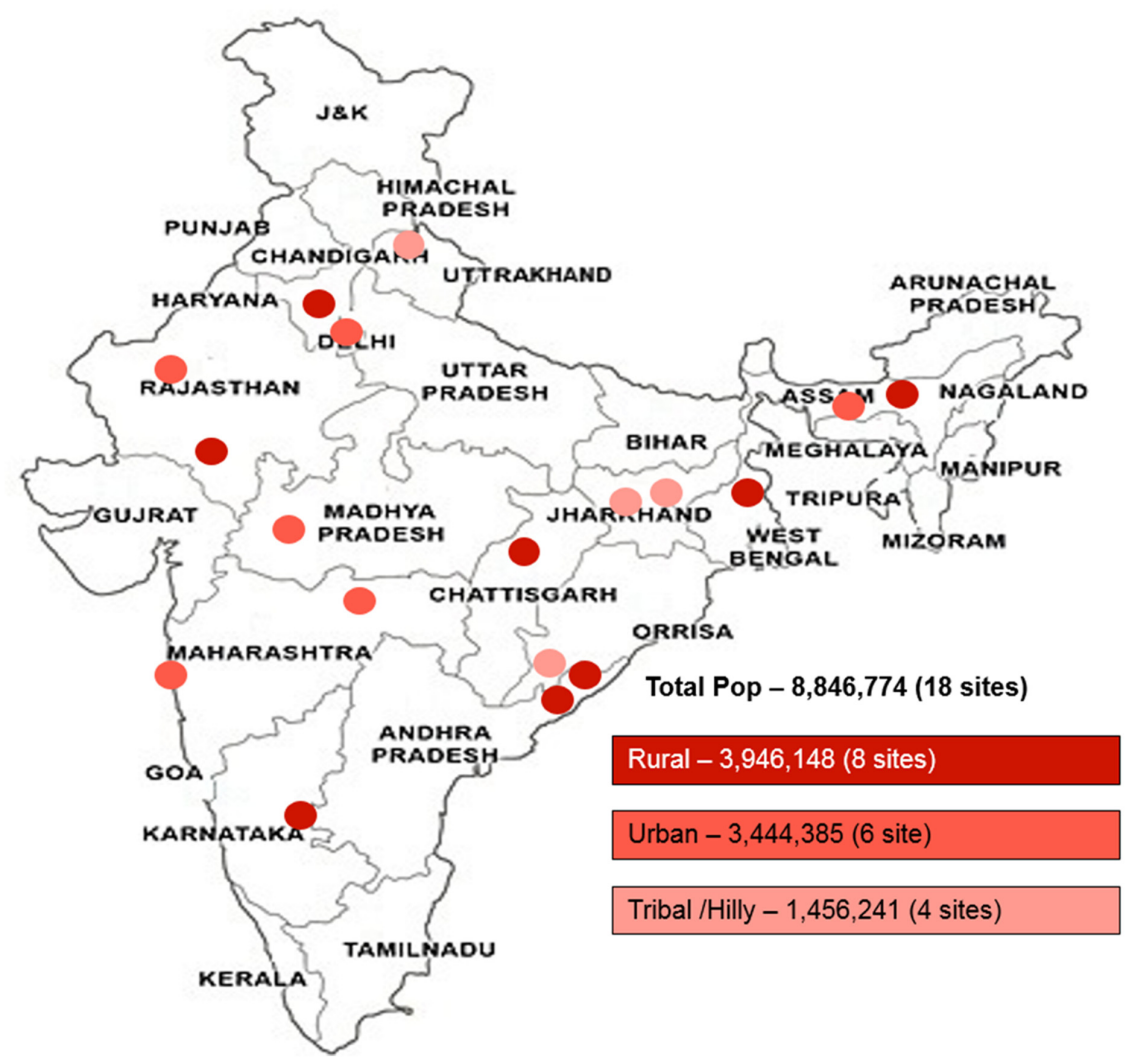
Geographical location of study treatment units and the demographic classification assigned to each project treatment unit site.

India’s RNTCP services cover a population of 1.2 billion. The program has subdivided the country into 662 district TB programme units and 2,698 sub-districts that are referred to under RNTCP as Tuberculosis units (TUs). Each TU is structured to include a population of approximately 0.5 million. Each TU has 4–6 designated sputum smear microscopy centers (DMCs), with each DMC covering approximately a population of 0.1 million. Each DMC is linked to 3–5 primary health centers that refer presumptive TB patients to the respective DMC. This study was conducted in 18 selected TUs. These study TUs were selected by a national committee purposively to reflect a broad diversity of settings relevant for TB control practice with regard to geographic area, urban/rural composition, TB burden, and also based on the availability of free treatment for patients diagnosed with rifampicin resistance. Among the 18 study TUs, 8 study TUs were in rural areas covering a population of 3.9 million; 6 study TUs were in urban areas accounting for a population of 3.4 million; and 4 study TUs were in tribal and hilly areas, i.e. hard to access and sparsely populated areas [6], covering a population of 1.5 million populations (Fig 1). Altogether, these 18 study TUs accounted for 8.8 million people having access to TB diagnostic services through 99 DMCs and their corresponding linked health facilities.

### Patient enrollment and definitions

All presumptive pulmonary TB patients and presumptive DR-TB patients attending public health facilities within the selected study TU were enrolled in the study over the study period.

Presumptive pulmonary TB patients [7] were defined as individuals with symptoms suggestive of pulmonary TB (cough of 2 weeks or more, with or without other symptoms) who had sputum samples tested for TB. Similarly, presumptive pediatric PTB cases includes children presenting with fever and/or cough for ≥2 weeks, with or without weight loss or no weight gain, or showing neurological symptoms like irritability, refusal to eat, headache, and vomiting.

Bacteriologically confirmed pulmonary TB (PTB) [8] *cases* were defined as patients with a positive test result for TB. During the baseline phase sputum smear microscopy was performed on two specimens and a test was positive if at least one of two smears was positive for acid fast bacilli by Ziehl-Neelsen (ZN) microscopy. During the intervention phase one sputum specimen was tested and a test was positive if it was positive by the Xpert MTB/RIF assay, or if it was sputum smear positive in case the Xpert MTB/RIF assay result was a test failure (either invalid, error or no results) or unavailable. Patients with a negative Xpert MTB/RIF result, but positive sputum smear microscopy were excluded in the outcome ‘bacteriologically confirmed PTB’ since under routine use of Xpert, patients with negative Xpert results would not be tested with sputum smear microscopy.

Clinically diagnosed pulmonary TB [8] *cases* are defined as cases that do not fulfill the criteria for bacteriological confirmation but are diagnosed with active TB by a treating physician—using a standardized programmatic diagnostic algorithm, which incorporates chest X-ray, antibiotic trial, repeat smear microscopy and clinical evaluation of symptoms—and are initiated on TB treatment, as evidenced by registration in a RNTCP treatment register.

Pulmonary TB *cases* were defined as any bacteriologically confirmed or clinically diagnosed case of TB involving the lung parenchyma or the tracheo-bronchial tree. A pulmonary TB case without prior treatment for TB (or less than 1 month of treatment) was considered a new case. A TB case that had more than one month of anti-TB treatment in the past was defined as previously treated [7].

Presumptive DR-TB patients were defined as already diagnosed pulmonary TB cases based on smear-microscopy referred for drug susceptibility testing (DST) because of an elevated risk of drug-resistant TB. National programme guidelines define high-risk TB cases as those TB cases with previous history of anti TB treatment, TB cases on treatment with positive sputum smear result at any follow up smear examination, diagnosed TB cases with HIV-co-infection and all pulmonary TB cases who are contacts of a known MDR TB case [9]. The definition of presumptive DR-TB cases was maintained in the intervention phase as all cases with high-risk for DR-TB even though everybody was tested for rifampicin resistance with Xpert MTB/RIF.

Rifampicin resistant TB cases were defined as bacteriologically confirmed TB cases with indication of rifampicin resistance on one or more of the following assays: Xpert MTB/RIF, line probe assay (LPA) or phenotypic DST.

### Study Design

This study was implemented in two phases a baseline and an intervention phase from March 2012 to December 2013. The baseline phase served as a reference prior to implementation of Xpert MTB/RIF. Fourteen out of 18 study TUs collected 2–5 months of baseline information. In the intervention phase, Xpert MTB/RIF was offered to all presumptive TB patients and presumptive DR-TB patients. As per the study protocol, 4 study TUs bypassed baseline phase and began the intervention phase directly to generate early experience on installation, infrastructure, and training needs (Fig C in S1 File). No mobilization efforts were built in the study design to influence testing or referrals of presumptive TB patients, either from the private or the public sector.

In the baseline phase, programmatic data including patients demographic profile, smear microscopy results, culture DST information in case of presumptive DR-TB cases and treatment initiation and follow up status for TB and DR TB cases, was captured In this phase, the existing RNTCP diagnostic algorithm (Fig A in S1 File) was followed. Accordingly, all persons who were identified as presumptive TB patients in health facilities were referred to DMCs where 2 sputum samples were collected and examined for microscopy. For patients, who were suspected to have DR TB and required a DST, two additional sputum samples were collected and sent to the nearest regional RNTCP DST laboratory for DST using LPA and/or phenotypic DST. In this phase, DST was offered selectively to diagnosed TB cases with high risk of drug resistance as per existing national programme guidelines on presumptive DR-TB patients.

During the intervention phase, one Xpert MTB/RIF laboratory was established in each TU, at an existing DMC and one or two, 4-module machine was installed. These laboratories have basic facilities for sputum smear microscopy in terms of infrastructure and human resources. All the patients suspected of having TB and DR TB that accessed services at the DMCs within the study TU (irrespective of place of residence) were enrolled in the study. Two sputum specimens, spot and morning samples were collected at the DMC and transported to the respective Xpert MTB/RIF lab, where Xpert MTB/RIF and ZN smear microscopy were done. Xpert MTB/RIF was always performed on the 1^st^ received sample (Fig B in S1 File).

In the intervention phase, treatment of TB was initiated based on Xpert MTB/RIF results in line with project diagnostic algorithm (Fig B in S1 File). Suspects with a negative result for TB on Xpert MTB/RIF and a positive smear result on microscopy were managed based on results of smear microscopy. These patients were asked to provide an additional sputum specimen that was sent for solid or liquid culture to confirm the diagnosis of TB. Patients diagnosed as rifampicin-resistant on Xpert MTB/RIF were to have an additional specimen collected prior to treatment initiation and sent to regional RNTCP DST laboratories for solid/liquid media DST and line probe assay (LPA) to confirm Xpert MTB/RIF results. Due to programmatic limitations, only a sample collected shortly after treatment initiation (and not a pretreatment sample) was available for some patients. Treatment of rifampicin resistant TB was initially based on Xpert MTB/RIF rifampicin resistant result. If conventional DST results were discrepant from Xpert MTB/RIF and/or LPA result, results of conventional DST were used for clinical decision-making.

Linkage to TB treatment for all diagnosed patients was ensured by capturing information on treatment initiation and addressing possible gaps in treatment initiation. As per the existing RNTCP or national programme, a TU maintains records and undertakes active management and follow-up of diagnosed TB cases that reside within the TU and are initiates them on treatment at facilities within the TU. Hence, collecting information on treatment initiation of all diagnosed TB cases that resided outside the study TU was not within the scope of the current study. However, if such a case was diagnosed the programme officials in the relevant TU, (where the diagnosed case resides) were informed on the details of the case by email and SMS.

### Laboratory Methods

Sputum smear microscopy was conducted by the existing public sector laboratory technicians who followed the RNTCP smear microscopy guidelines with all functional components of quality assurance (QA) in place [10]. Testing on Xpert MTB/RIF assay was conducted by the existing public sector laboratory technicians. A one day training was given to the laboratory technicians on standard operating procedure recommended by the manufacturer for Xpert MTB/RIF testing [11]. The laboratory staff was trained on RNTCP guidelines and project aspect including data collection by the project team consisting of program manager and project coordinators. The technical resource person from Cepheid provided training on using GeneXpert and related aspects with hands on experience on performing GeneXpert testing. To ensure quality of results of Xpert MTB/RIF assay, all equipment was validated using GLI Xpert MTB/RIF validation panels. In case of 'error', 'invalid', 'no result' or 'rifampicin indeterminate' results the tests were repeated on the second sample [12].

Solid /liquid media DST and line probe assay were performed for patients diagnosed as rifampicin-resistant TB on Xpert MTB/RIF. Confirmatory DST was performed only at RNTCP accredited culture and DST laboratories following programme guidelines [13–14]. At 17 of these laboratories, DST was performed on Lowenstein-Jensen (LJ) medium and at one laboratory DST was performed on BACTEC MGIT 960 broth culture system. Genotype MTBDR *plus* Version 2 kit was used to perform LPA at all these laboratories.

### Data management and statistical analysis

Data was collected for all presumptive pulmonary TB and presumptive DR-TB patients using standardized case report forms (CRFs) by the RNTCP staff working at DMCs and study TUs. Data from CRFs was entered via a secure, web-based MIS (Management Information System) by the site staff. Quality of data was ensured by regular scrutiny of CRFs using cross validation against programme records by project supervisors. Data were analysed using STATA version 12.

The main outcomes of the analysis were:

1. The effect of Xpert MTB/RIF implementation on the notification of bacteriologically confirmed PTB and all PTB, expressed per 100,000 population per year (hereafter called the notification rate),
2. The effect of Xpert MTB/RIF implementation on the proportion of bacteriologically confirmed PTB and all PTB out of the numbers of persons tested for presumptive TB,
3. The positive predictive value of detection of rifampicin resistance by Xpert MTB/RIF.

To calculate notification rates, the number of bacteriologically confirmed PTB and of all PTB notified per study area or TU were divided by the population size residing within the respective TU, and multiplied by the duration of the baseline and intervention phase, respectively, to account for variable duration of data collection in each TU and per study period. The population for each TU as of 1 January 2012 was estimated by the RNTCP based on 2010 census data and growth projections. Population size during the study period was also adjusted for population growth, assuming linear growth during monthly intervals, summing up to an annual growth of 1.27% [15].

For each of the 14 TUs that collected both data in the baseline and intervention phase, PTB notification rates and the annualized number of patients tested per 100,000 were adjusted for any changes in the distribution of age, sex, TB treatment history, and type of provider (public or private) requesting the test in the presumptive PTB patient population between the baseline and intervention phase by standardizing the patient population tested in the intervention phase against the patient population in the baseline phase using inverse probability weighting. The reported notification rates are calculated as means of the 14 TU specific notification rates. The effect of Xpert implementation on the notification rates was estimated using a random-effect negative binomial regression model to control for clustering at the study site level.

It is possible that the underlying population from which the presumptive PTB and DR-TB patients arose differed between the baseline and intervention phase, e.g. through increased referral from outside of the TU. Such changes could not be assessed directly since information on residency within or outside the TU was not available for presumptive TB patients. Therefore, in addition, the effect of Xpert implementation on the proportion of bacteriologically confirmed and all PTB diagnosed among presumptive TB patients was estimated in the same 14 sites with data from the baseline and intervention phase. The case detection proportion is more robust to changes in the underlying population than the notification rate. Risk ratios were calculated reflecting the effect of Xpert MTB/RIF implementation on proportions of PTB diagnosed among presumptive TB patients. Risk ratios were estimated using log-binomial regression models with robust standard errors to account for clustering defined at the TU level. The effects of the same covariates (gender, age distribution, history of TB treatment, type of referring provider) plus TU area characteristic were examined in this model to adjust for potential confounding due to changes in distribution of these covariates among presumptive PTB patients between the baseline and intervention period.

To calculate positive predictive values (PPV) of a positive result for rifampicin resistance in Xpert MTB/RIF against phenotypic DST and against LPA as the reference standard, data collected during Xpert MTB/RIF implementation in all 18 study TUs were used.

Finally, the proportion of bacteriologically confirmed PTB cases and rifampicin resistant TB cases being initiated on TB treatment was assessed for all 18 TUs among PTB cases residing within the study TU.

### Additional analyses

The current manuscript focuses on describing our assessment of the impact of Xpert MTB/RIF implementation on the diagnosis of TB and DR-TB cases in programmatic settings. The feasibility and performance of Xpert MTB/RIF, with regards to test failure rates, is the subject of a separate publication [12]. In addition, a cost-effectiveness analysis is being performed and will be published separately.

### Ethical considerations

The study protocol was approved by the Institution Ethics Committee of the National Tuberculosis Institute, Bangalore, India. Structured informed consent forms were used for obtaining written consent from all subjects enrolled in the study. Before taking consent, patients were informed about the study in vernacular language by the trained staff. For illiterate patients, consent was taken in presence of literate witness; similarly written consent for the children less than 18 years of age was obtained from the parents / guardians accompanying them. Approval for the study was granted by the Central TB Division, Ministry of Health and Family Welfare, Government of India.

## Results

**Fig 2:**
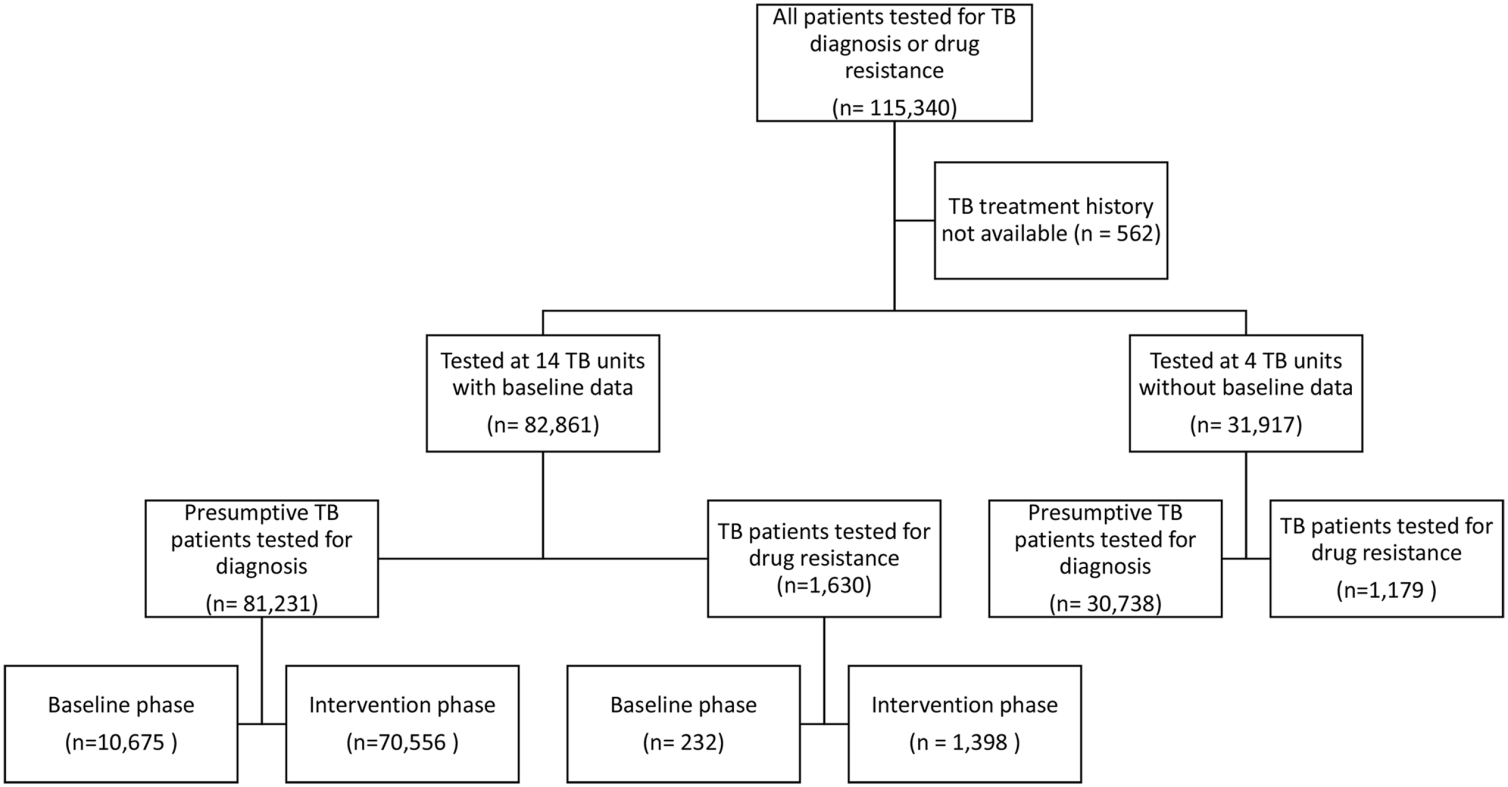
Enrollment of presumptive tuberculosis and drug resistant tuberculosis cases in baseline and intervention period of the study.

A total of 115,340 patients were approached under the study and all patients agreed to be enrolled across 18 study TUs. We excluded 562 (0.5%) from the analysis because of missing treatment history information. Of the remaining 114,778 patients, 111,969 (97.6%) were presumptive TB patients. In the 14 TUs with both baseline and intervention data, 10,675 (97.9%) of subjects enrolled in the baseline phase were presumptive TB patients and 232 (2.1%) were presumptive DR-TB patients. In the intervention phase 70,556 (98.1%) presumptive TB patients and 1,398 (1.9%) presumptive DR-TB patients were enrolled (Fig 2).

The demographic characteristics of the presumptive TB patients tested in the baseline and intervention period of the study in these 14 study TUs are shown in Table 1. The distributions of age and sex were very similar in the intervention phase. However, 6.8% of patients during the baseline phase and 16.9% in the intervention phase had prior history of anti TB treatment. The urban TUs contributed 33.8% of patients during the baseline and 40.8% during the intervention phase, while the contribution of the TUs from tribal/hilly areas decreased from 27.2% to 20.5%.

**Table 1:** Characteristics of persons tested for pulmonary tuberculosis by study phase, at the 14 study treatment units with data collection both in the baseline and intervention phase.

	Baseline		Intervention		Total
	N	%	N	%	
**Total**	10,675	100%	70,556	100%	81,231
**Age category (Years)**					
<15	428	4.0%	2,570	3.6%	2,998
15–29	2,864	26.8%	17,729	25.1%	20,593
30–44	3,274	30.7%	20,373	28.9%	23,647
45–59	2,333	21.9%	16,821	23.8%	19,154
60–74	1,574	14.7%	11,609	16.5%	13,183
>75	202	1.9%	1,454	2.1%	1,656
**Gender**					
Female	3,842	36.0%	24,820	35.2%	28,662
Male	6,833	64.0%	45,736	64.8%	52,569
**Past history of anti TB treatment**					
No	9,951	93.2%	58,634	83.1%	68,585
Yes	724	6.8%	11,922	16.9%	12,646
**Geographical Distribution**					
Urban	3,609	33.8%	28,761	40.8%	32,370
Rural	4,165	39.0%	27,322	38.7%	31,487
Tribal/Hilly	2,901	27.2%	14,473	20.5%	17,374
**PTB diagnosis**					
No TB	8,674	81.3%	54,873	77.8%	63,547
Xpert-negative; smear-positive	NA		97	0.1%	97
Bacteriologically confirmed PTB					
Xpert-positive; smear-negative/NA	NA		4,524	6.4%	4,524
Xpert-positive; smear-positive	NA		9,600	13.6%	9,600
Xpert-Indeterminate; smear-positive	NA		13	0.0%	13
Xpert-NA; smear-positive	1,532	14.4%	162	0.2%	1,694
Clinically-diagnosed PTB	469	4.4%	1,287	1.8%	1,756
**Total**	10,675	100%	70,556	100%	81,231

Abbreviations: ART = antiretroviral treatment; NGO = non-governmental organization; PTB = pulmonary tuberculosis; Bacteriologically confirmed PTB = All bacteriologically diagnosed (smear and or Xpert MTB/RIF) PTB cases; sm = sputum smear microscopy; Xp = Xpert MTB/RIF; neg = negative; pos = positive; na = not available

### Effect of Xpert MTB/RIF implementation on pulmonary TB case notification

The adjusted all PTB case notification rate per 100,000 population years increased from 116 (95% Confidence Interval [CI] 83–149) in the baseline phase to 134 (CI 100–168) in the intervention phase (Table 2). The adjusted incidence rate ratio (aIRR) was 1.16 (CI 1.01–1.33), representing a 16% (CI 1–33) increase in the case notification rate of all PTB. The adjusted bacteriologically diagnosed case notification rate per 100,000 population years increased from 90 (CI 62–177) in baseline phase to 123 (CI 90–155) in the intervention phase (Table 2). The aIRR for the bacteriologically confirmed PTB case notification rate was 1.39 (CI 1.18–1.64).

**Table 2:** Number of presumptive TB patients tested, population time at risk, tuberculosis cases diagnosed, and incidence risks during the baseline and intervention periods at each Treatment Unit, and the incidence risk ratios comparing the intervention period to the baseline period.

	BASELINE PERIOD							INTERVENTION PERIOD							IRR		
TU	Presumptive TB patients tested	Person-years*	IR of presumptive TB patients tested per 100,000 population per year	All PTB cases diagnosed	IR of all PTB diagnosed cases per 100,000 population per year	Bact+ PTB cases diagnosed	IR of bact+ PTB cases per 100,000 population per year	Presumptive TB patients tested	Person-years*	IR of presumptive TB patients tested per 100,000 population per year§	All PTB cases diagnosed§	IR of all PTB diagnosed cases per 100,000 population per year§	Bact+ PTB cases diagnosed§	IR of bact+ PTB cases per 100,000 population per year§	IRR—presumptive TB cases tested	aIRR—all PTB§	aIRR bact+ PTB§
**Urban**																	
Site 1	1001	121746	822	212	174	192	158	7353	887712	811	1549	174	1260	142	0.99	1.00	0.90
Site 2	1003	149529	671	114	76	76	51	9607	1069816	890	1328	124	1077	101	1.33	1.63	1.98
Site 3	493	90525	545	79	87	55	61	2745	642169	406	758	118	673	105	0.74	1.35	1.72
Site 4	1112	137344	810	288	210	216	157	9056	1284902	688	2854	222	2695	210	0.85	1.06	1.33
**Rural**																	
Site 5	510	187540	272	108	58	86	46	2796	604618	447	729	121	703	116	1.64	2.09	2.53
Site 6	761	119713	636	113	94	60	50	6774	880634	761	1313	149	1171	133	1.20	1.58	2.65
Site 7	1287	129361	995	278	215	181	140	10312	979011	1044	2200	225	2077	212	1.05	1.05	1.52
Site 8	433	124201	349	78	63	63	51	1556	483836	318	291	60	275	57	0.91	0.96	1.12
Site 9	591	208846	283	124	59	79	38	3030	1161870	259	548	47	474	41	0.92	0.79	1.08
Site 10	583	86060	677	63	73	61	71	2854	741209	373	635	86	628	85	0.55	1.17	1.19
**Tribal/Hilly**																	
Site 11	999	133933	746	223	167	192	143	3806	508877	739	1006	198	955	188	0.99	1.19	1.31
Site 12	459	49381	930	59	119	49	99	1980	285579	680	321	112	292	102	0.73	0.94	1.03
Site 13	333	113172	294	78	69	58	51	2704	736074	347	478	65	411	56	1.18	0.94	1.09
Site 14	1110	116323	954	184	158	164	141	5983	756570	771	1352	179	1317	174	0.81	1.13	1.23
Total (95%CI) ^	10675	1767676	642	2001	116 (83–149)	1532	90 (62–117)	70556	11022876	610	15363	134 (100–168)	14006	123 (90–155)	1.00	1.16 (1.01–1.33)	1.39 (1.18–1.64)
												(100–168)			(0.87–1.14)		

aIRR = adjusted incidence ratio; bact+ = bacteriologically confirmed; RIF = rifampicin; IR = incidence risk; IRR = incidence rate ratio; PTB = pulmonary tuberculosis

* Number of person-years is calculated from TU population size multiplied by time in baseline or intervention period. The population size is adjusted for 1.27% annual population growth during the study period (adjusted on a monthly basis, assuming linear increase)

^§^ Adjustment for difference in the distribution of characteristics of presumptive TB patients between intervention and baseline by inverse probability weighting (standardization of the intervention patient population to the baseline patient population)

^^^ IR and IRR with 95% Cis are calculated based on cluster-averaged means

The IR of all PTB and bact+ PTB is referred to in the manuscript text as the case notification rate

### Effect of Xpert MTB/RIF implementation on proportion of presumptive PTB cases diagnosed with TB

Of 10,675 presumptive TB patients tested during the baseline phase, 1,532 (14.4%) were smear-positive and an additional 469 (4.4%) patients were clinically diagnosed with PTB (Table 1). Of 70,556 patients tested during the intervention phase, 14,299 (20.3%) had a bacteriologically confirmed PTB diagnosis and an additional 1,287 (1.8%) were clinically diagnosed with PTB. Of the 14,124 patients with a positive Xpert MTB/RIF result 4,524 (32.0%) were smear-negative. The number needed to test with Xpert to diagnose one additional smear-negative TB case was 13.4.

Table 3 shows the proportion of patients diagnosed with bacteriologically confirmed PTB among all patients tested for TB diagnosis, stratified by age, gender, history of TB treatment, referring provider and area. The adjusted risk ratio (aRR) was 1.33 (CI 1.16–1.52), indicating an average increase in the proportion of bacteriologically confirmed TB cases among persons tested during the intervention phase of 33% (CI 16–52), compared to the baseline. The increase in the proportion of patients diagnosed was highest among those aged 60 years and older and for patients from the rural area. Among patients with no previous history of TB treatment, the increase was slightly lower (RR 1.31, CI 1.11–1.54) than among those treated previously (RR 1.44, CI 0.96–2.16), although not significantly. The aRR between baseline and intervention phase for the detection of all PTB cases, out of the total presumptive TB patients was 1.11 (CI 1.03–1.21) (Table A in S1 File). Additional sensitivity analyses are described in Annexure 1 in S1 File.

**Table 3:** Proportion of bacteriologically confirmed pulmonary tuberculosis cases diagnosed at the 14 study treatment units with data collection both in the baseline and intervention phase.

	Baseline			Intervention			Risk Ratio of the effect of the intervention in each stratum, adjusted for clustering at TU level*			Adjusted Risk Ratio of the Effect of the Intervention in Each Stratum^#^		
Characteristic	Suspects	Bact+ PTB	Row %	Suspects	Bact+ PTB	Row %	RR	95% CI		RR	95% CI	
**Total** ^**$**^	10,675	1,532	14.4%	70,556	14,299	20.3%	1.41	1.22	1.63	1.33	1.16	1.52
**Age category (Years)**												
<15	428	22	5.1%	2,570	196	7.6%	1.48	0.86	2.55	1.46	0.86	2.50
15–29	2,864	480	16.8%	17,729	4,300	24.3%	1.45	1.22	1.71	1.33	1.15	1.55
30–44	3,274	490	15.0%	20,373	4,414	21.7%	1.45	1.25	1.67	1.35	1.17	1.54
45–59	2,333	362	15.5%	16,821	3,324	19.8%	1.27	1.09	1.48	1.22	1.03	1.43
60–74	1,574	167	10.6%	11,609	1,917	16.5%	1.56	1.33	1.83	1.48	1.25	1.75
> = 75	202	11	5.4%	1,454	148	10.2%	1.87	0.87	4.02	1.72	0.79	3.72
**Gender**												
Female	3,842	414	10.8%	56,584	11,406	20.2%	1.41	1.12	1.77	1.34	1.07	1.68
Male	6,833	1,118	16.4%	13,972	2,893	20.7%	1.41	1.24	1.60	1.33	1.17	1.50
**Past History of anti TB treatment**												
No	9,951	1,370	13.8%	58,634	10,496	17.9%	1.30	1.10	1.54	1.31	1.11	1.54
Yes	724	162	22.4%	11,922	3,803	31.9%	1.43	0.94	2.16	1.44	0.96	2.16
**Type of referring provider**												
Public	8,926	1,279	14.3%	56,584	11,406	20.2%	1.41	1.19	1.66	1.32	1.12	1.56
Other	1,749	253	14.5%	13,972	2,893	20.7%	1.43	1.22	1.68	1.34	1.16	1.54
**Geographical distribution**												
Urban	3,609	539	14.9%	28,761	5,842	20.3%	1.36	0.98	1.89	1.21	0.93	1.57
Rural	4,165	530	12.7%	27,322	5,408	19.8%	1.56	1.33	1.81	1.52	1.31	1.76
Tribal/Hilly	2,901	463	16.0%	14,473	3,049	21.1%	1.32	1.09	1.59	1.25	1.06	1.47

^$^n = 81,231; 562 patients with missing values for history of anti-TB treatment were excluded.

*Robust standard errors to adjust for clustering at TU level

^#^ Adjusted for clustering at TU level, AND adjusted for age group, sex and past TB history.

Abbreviations: Suspects = number of presumptive pulmonary TB patients tested; All PTB = All diagnosed cases of pulmonary tuberculosis; Bact+PTB = bacteriologically confirmed pulmonary tuberculosis; RR = adjusted relative risk ratio; 95% CI = 95% confidence interval

### Effect of Xpert MTB/RIF implementation on rifampicin-resistant TB case detection

In the baseline phase, when DST was selectively done on samples from presumptive DR-TB patients, 31 (0.3%) rifampicin resistant cases were identified among 10,907 presumptive TB and DR-TB patients combined. In the intervention phase, at the same 14 TUs, this increased to 1.7% (1190/71,954), a more than 5 fold increase with universal upfront testing for rifampicin resistance on Xpert MTB/RIF. The notification rate of rifampicin resistant TB cases increased from 1.9 during the baseline to 9.9 per 100,000 during the intervention phase. An aIRR was not obtained due to TUs with zero rifampicin resistant cases during the baseline phase (Table B in S1 File).

Among all patients tested in the intervention phase across all 18 study TUs, overall 2,765 (12.2%) of 22,686 Xpert MTB/RIF positive PTB cases had a rifampicin resistant result on Xpert, of whom 464 (16.8%) were among PTB cases classified as presumptive DR-TB cases. The remaining 2,301 (83.2%) were among PTB cases detected among presumptive TB patients. In total, 63.5% (1,460/2,301) of rifampicin resistance PTB cases were detected among those with a previous history of TB treatment (24.1% prevalence of rifampicin resistance in this group; Table 4). Among new PTB cases 5.8% (841/14,539) had a positive result for rifampicin resistance with Xpert MTB/RIF.

**Table 4:** Numbers and proportion of tuberculosis patients with rifampicin resistance detected with Xpert MTB/RIF across all 18 study TUs in intervention phase.

Category	Confirmed PTB Cases on Xpert	Number Detected with additional Rifampicin resistance	Xpert RIF resistance prevalence
**Presumptive TB patients**	20587	2301	11.2%
** New**	14539	841	5.8%
Xpert Positive; Smear-positive	9308	474	5.1%
Xpert positive; smear-neg/NA	5231	367	7.0%
** Previously treated**	6048	1460	24.1%
Xpert positive; Smear-positive	3837	979	25.5%
Xpert positive; smear-neg/NA	2211	481	21.8%
**TB patients who are DR-TB patients**	2099	464	22.1%
Xpert positive; Smear-positive	1642	403	24.5%
Xpert positive; smear-neg/NA	457	61	13.3%
**All Presumptive TB patients and DR-TB patients combined**	22686	2765	12.2%

Abbreviations: PTB = All diagnosed cases of pulmonary tuberculosis; DR-TB patients = number of presumptive pulmonary drug resistance TB patients tested

Of the total 2,765 rifampicin resistant cases, additional samples for confirmatory DST for 2,059 (74.5%) cases was collected and sent to culture and DST lab. The samples for 706 (25.5%) could not be collected due to various program limitations including initial default cases, not being traced and other field limitations. However, it is difficult to comment on how much each factor has attributed to each of the missing cases. At the time of study data closure confirmatory DST and/or LPA test results were available for 1,620 (78.7%) of 2,059 rifampicin resistance cases whose samples were sent to the lab. As a result of this study being conducted under uncontrolled programmatic conditions, only 1,055 (65%) of these had a phenotypic DST result. Major reasons for non-availability of DST results were contaminated cultures 48 (2.3%), no growth on culture possibly due to delayed collection (when already on 2nd line therapy) in 331 (16.1%), and study data closure prior to availability of culture DST results 524 (19%). Common reason for non-availability of LPA results 320 (11.6%) was Xpert MTB/RIF positive, smear negative specimen with no-growth on culture. An additional (140) 5.1% of the specimen had an invalid result on LPA. Compared to rifampicin resistance on phenotypic DST as the reference standard, the overall PPV of a positive Xpert MTB/RIF result was 94.7% (CI 91.3–98.1). The PPV in presumptive TB patients and in presumptive DR-TB patients were very similar, with overlapping 95% CIs (Table 5). Among presumptive TB cases without a history of TB treatment, the PPV was 90.6% (CI 84.9–96.4) compared to 95.9% (CI 92.5–99.3) for those previously treated. Similar PPV was observed as compared with LPA as a reference standard (Table 5).

**Table 5:** Positive predictive value (PPV) of a rifampicin resistance signal on Xpert MTB/RIF among presumptive TB patients tested, during the intervention phase at all 18 treatment units.

	PPV compared to LPA in patients with both an Xpert result and LPA result			95% CI§	PPV compared to Culture DST in patients with both an Xpert result and Culture DST result			95% CI§	PPV compared to either Culture DST and/or LPA in patients with both an Xpert result and Culture DST and/or LPA result*			95% CI§
Category	Number of results available	LPA Rif Res	PPV		Number of results available	Culture DST Rif Res	PPV		Number of results available	LPA/Culture DST Rif Res	PPV	
Presumptive TB patients												
New	368	336	91.3%	88.8–93.9	267	242	90.6%	84.9–96.4	439	402	91.6%	89.3–93.9
Previously treated	847	808	95.4%	93.9–96.9	661	634	95.9%	92.5–99.3	954	918	96.2%	94.5–98.0
All	1,215	1,144	94.2%	92.6–95.7	928	876	94.4%	90.8–98.0	1,393	1,320	94.8%	93.1–96.4
TB patients who are DR-TB suspects												
	212	198	93.4%	89.3–97.5	127	123	96.9%	93.5–100.0	227	215	94.7%	89.5–99.9
Presumptive TB patients and DR-TB suspects combined												
	1427	1342	94.0%	92.4–95.6	1055	999	94.7%	91.3–98.1	1620	1535	94.8%	92.9–96.6

*If results are available for both phenotypic DST and LPA, a rifampicin resistance result on at least one of those is considered as confirmatory

^§^Adjusted for clustering at site level using robust standard errors

Abbreviations: PPV = Positive Predictive Value; LPA = Line probe assay; Culture DST = phenotypic drug susceptibility testing; PTB = pulmonary TB; Rif Res = Rifampicin resistance; DR suspect = number of presumptive pulmonary drug resistance TB patients tested, TB suspect = number of presumptive pulmonary TB patients tested

### Initiation of bacteriologically confirmed and rifampicin resistant PTB cases on TB treatment

By definition all clinically diagnosed patients started treatment. Treatment initiation records were analyzed for bacteriologically confirmed TB cases without evidence of rifampicin resistance among patients categorized as presumptive TB patients. In the baseline phase, 1,022 (69%) resided within the study TUs and evidence of treatment initiation could be verified for 928 (90.8%). In the intervention phase, 11,905 (64%) resided in the TUs and 10,722 (90.1%) initiated treatment. Of 2,765 rifampicin resistant TB patients detected in the intervention phase, 1183 (42%) resided within the study TUs and treatment initiation could be verified for 974 (82%). Second-line drug treatment at the time of data collection closure had been started for 876 (90%) of these patients. Common reasons for initial loss to follow-up include death prior to treatment initiation, inability to trace cases after diagnosis, refusal of treatment, loss to follow-up within one month of treatment initiation without being registered in the TB register. However, due to the pragmatic nature of the study, data on date of treatment initiation was not available for 277 (2.6%) PTB cases and time to treatment initiation could not be analyzed. Registered treatment initiation may be underestimate as some of these cases for which information could not be verified by the study team could be under evaluation prior to initiation of second-line treatment. Also patients lost to follow-up could have returned on treatment. However, this could not be verified.

## Discussion

In this large-scale demonstration study across diverse settings in India, Xpert MTB/RIF deployment as the initial TB diagnostic test in public health facilities, significantly increased TB case finding. Substitution of smear microscopy by Xpert MTB/RIF on average increased the rate of TB case notification by 16% and of bacteriologically confirmed TB case notification by 39%. Similarly, the proportion of presumptive TB patients with a TB diagnosis increased by 11% for all forms of pulmonary TB, and by 33% for microbiologically confirmed TB cases, taking confounding by age, sex and history of prior TB treatment into account. These findings are similar to those reported from South Africa and Brazil and underscore the potential benefit of using a rapid, high sensitivity TB diagnostic test [16–17].

As a consequence of the increased bacteriological confirmation among those tested, we noted a decrease in the proportion of clinically diagnosed cases with upfront testing on Xpert MTB/RIF in the intervention phase (crude percentages 4.4% and 1.8%) (Table 1). As per the national guidelines, clinical diagnosis of a TB case in the absence of bacteriological evidence requires the individual to undergo a two week antibiotic trial and chest X-ray examination along with repeat smear microscopy. This decrease in the proportion of clinically diagnosed TB cases is indicative of the potential benefit in terms of reduced attrition of presumptive TB cases, time to diagnosis, and cost of diagnosis, both to the patient as well as to the health system. However, data both from South Africa and Brazil also showed an increase in bacteriologically confirmed TB but no increase in persons started on treatment [21]. Furthermore, the South African data also did not show an effect on initial loss to follow-up within 4 weeks.

There was also a substantial increase in the detection of rifampicin resistant TB cases by offering a rapid drug resistance test to all presumptive TB patients, using Xpert MTB/RIF, instead of only selectively offering conventional DST to already diagnosed TB patients with a high risk of having drug resistance. Similar findings were documented in the past in studies conducted in South Africa, Uganda and India with Xpert MTB/RIF [18]. In our study, almost one third of rifampicin resistant TB cases were detected among Xpert MTB/RIF positive TB cases with no prior history of TB treatment. This finding demonstrates the potential impact of extending universal DST to all presumptive TB cases under routine programme conditions in improving case finding of TB as well as rifampicin-resistant TB, particularly in areas where drug-resistance in treatment naïve cases is of substantial concern.

The positive predictive value for detecting rifampicin resistance using Xpert MTB/RIF was very high, overall (94.8%) and even among new PTB patients with no history of anti-TB treatment (91.6%), of whom 5.8% had a rifampicin resistant Xpert MTB/RIF result. This overall PPV of rifampicin resistant in our study was slightly higher than previously reported from South Africa (89.7%) [19]. In India, where the prevalence of rifampicin resistance in new TB cases is estimated to be around 3% [9], these data imply that treatment for MDR-TB could arguably be initiated with confidence in any patient with rifampicin-resistance results detected by Xpert MTB/RIF, regardless of prior treatment history. Repeat drug susceptibility testing for previously-treated TB patients with rifampicin-resistant results from Xpert for MDR-TB (PPV 96.2%), as recommended by current WHO guidelines, may not be required. In the absence of alternative rapid diagnostics, this PPV of 91.6% in TB cases with no history of anti TB treatment, even though not ideal makes a strong case for initiating second line treatment for cases found rifampicin resistant on Xpert MTB/RIF, with in parallel confirmatory DST on liquid culture as gold standard. Data on false positivity in this group of treatment naïve cases can be monitored over a period of time for appropriate future policy decision making.

## Limitations

The current study was carried out in uncontrolled programmatic field settings, with key project intervention being upfront Xpert MTB/RIF testing for all presumptive TB and DR-TB patients. The duration of baseline phase in the TUs with baseline data was a minimum of 2 months. For pragmatic reasons, the exact date of initiation of project baseline and intervention phase was based on site preparedness and was therefore not be randomized. As with any non-randomized before-after comparison, the results may suffer from bias that cannot fully be adjusted for at the analysis stage. A phased implementation design with randomly assigned starting dates of the intervention spread over a longer time period would have allowed for more balanced sample sizes between baseline and intervention period, and adjustment for possible temporal trends. The limited sensitivity analysis that could be done, adjusting for calendar period in the baseline period, did not reveal an underestimation of the effect of the intervention. It should be noted that our findings may be specific to the situation in the public sector in India and that the results may not be generalizable to other settings and other patient populations [22].

The effect of the intervention on case detection may have been affected by changes between the baseline and intervention phase in the number and characteristics of patients tested, as suggested by the higher proportion of presumptive TB patients with prior history of TB treatment and by somewhat lower proportions of TB patients residing in the study TUs during the intervention phase. We hypothesize that the increase in previously treated presumptive cases at least in part may be the result of increased referrals from neighboring TUs. For this reason the study team chose to also present the proportion of TB cases detected out of all patients tested as the outcome for case detection, in addition to numbers per 100,000 persons per year. The analysis of the proportion of cases detected allowed for adjustment for confounding due to changes in patient characteristics, and clustering at TU level. Further, the effect on case notification included adjustment for TU population growth, standardization of patient characteristics, and variation between sites. Although the crude number of patients tested per month increased slightly during the intervention phase, after the adjustments, the annualized rates of patients tested per 100,000 population did not differ (aIRR 1.0) (Table A in S1 File). The results of both analyses point in the same direction, which supports an effect of Xpert MTB/RIF implementation on the detection of bacteriologically confirmed PTB. The observed effect on all PTB was smaller, and is less certain. This could be due to the fact that clinically diagnosed PTB cases were not confirmed by sputum culture and may be misclassified. Second, the proportion of clinically diagnosed TB patients is likely underestimated in our calculations, since treatment initiation in the absence of a bacteriological test for patients tested at the TU but initiating treatment outside the TU could not be ascertained. Since the proportion of patients with clinically diagnosed TB was greater in the baseline compared to the intervention, the difference in the detection of all PTB may be less than the aRR of 1.11 (CI 1.03–1.21) and aIRR of 1.16 (CI 1.01–1.33) that we calculated.

While 18 study TUs participated in the intervention phase of the study, only 14 study TUs participated in the baseline phase. One of the 4 study TUs, which participated only in the intervention phase (in urban slum setting), had a higher proportion of rifampicin resistance, both amongst presumptive TB and DR-TB patients (Table B in S1 File). If these 4 sites also would have collected baseline data, the observed effect on case detection of drug resistance might have increased, as well as the positive predictive value of rifampicin resistance detection by Xpert MTB/RIF.

Even though attempts were made to individually contact every diagnosed case, whose treatment initiation information could not be traced directly in RNTCP records, the data collection was incomplete, particularly for patients residing outside of the study TUs. Therefore, we had to limit treatment initiation data to patients residing in the study TUs. Captured treatment initiation for bacteriologically confirmed TB patients at RNTCP clinics was 90%, both in the baseline and intervention phase. Still, for 10% of cases treatment initiation could not be verified. Some of these cases could have approached a private provider for treatment and treatment initiation may actually be higher. A 10% loss to follow-up, nonetheless, is lower than reported in most other settings [20]. Second-line drug treatment initiation for rifampicin-resistant PTB cases could be verified for almost 80% at the time of study closure. This compares to 92% globally in 2012 [1]. Some of the cases still could be on the waiting list for second-line treatment. As per RNTCP guidelines, the patient who is diagnosed with rifampicin resistance needs to undergo pretreatment evaluation. This as well as the need for initial hospitalization in a referral center led to delays in treatment initiation.

## Conclusion

This large-scale demonstration provides a robust data on the potential for increased case finding of TB and DR-TB through routine use of a high sensitivity molecular diagnostic test for TB and DR-TB (Xpert MTB/RIF) in public sector medical services. Our observations may be useful in guiding the decisions on scale-up of Xpert MTB/RIF in high-burden settings. The present study is also the first large scale attempt to offer universal DST to all presumptive TB cases in the public sector in India, and demonstrates the potential impact of this strategy in case finding of rifampicin resistant TB cases, particularly in countries with high levels of rifampicin resistance even in treatment-naive patients.

## Supporting Information

S1 File Annexure A, Sensitivity Analysis. Table A, Proportion of all pulmonary tuberculosis cases diagnosed at the 14 study treatment units with data collection both in the baseline and intervention phase. Table B, Number of presumptive tuberculosis and presumptive drug-resistant tuberculosis patients tested, population-time at risk, and tuberculosis patients with a positive Xpert MTB/RIF positive signal for rifampicin resistance diagnosed during the baseline and intervention periods at each Treatment Unit, and the incidence risk ratios comparing the intervention period to the baseline period. Fig A, RNTCP diagnostic Algorithm. Fig B, Study diagnostic Algorithm. Fig C, Transition of sites from baseline to intervention phase of the study.
(DOCX)

S2 File (XLSX)
