# Supplementary material for: Use of Xpert MTB/RIF in Decentralized Public Health Settings and Its Effect on Pulmonary TB and DR-TB Case Finding in India
Source: PLoS One. 2015 May 21;10(5):e0126065. doi: 10.1371/journal.pone.0126065 (PMC4440647; doi:10.1371/journal.pone.0126065)

**Supporting information_S1 file**

**Annexure A Sensitivity analysis**

Trends in case notification could have been influenced by the short duration of the baseline phase, underlying secular trends, or the influx of patients from surrounding areas during either phase. In a post-hoc sensitivity analyses, we therefore adjusted for calendar time by means of adjustment for quarter of the year. As the baseline phase for most sites largely overlapped in calendar time in 2012 and did not cover quarter 4 (October-December), we could only include data from quarters 1-3 in this analysis. These sensitivity analyses led to very comparable results, both for case detection rates and case detection proportions, and both for all PTB and bacteriologically confirmed PTB. For instance, the increase in case detection proportion of bacteriologically confirmed TB was 1.36 (CI 1.19–1.55) in data from quarter 1-3 not adjusting for calendar time, and 1.39 (CI 1.22–1.57) with adjustment for calendar time.

**Table A:** **Proportion of all pulmonary tuberculosis cases diagnosed at the 14 study treatment units with data collection both in the baseline and intervention phase**

|  | **Baseline** | | | **Intervention** | | | **Risk Ratio of the effect of the intervention in each stratum, adjusted for clustering at TU level** | | | **Risk Ratio of the Effect of the Intervention in Each Stratum^#^** | | |
| --- | --- | --- | --- | --- | --- | --- | --- | --- | --- | --- | --- | --- |
| **Characteristic** | **Suspects** | **All PTB** | **Row %** | **Suspects** | **All PTB** | **Row %** | **RR** | **95% CI** | | **RR** | **95% CI** | |
| **Total** | 10,675 | 2,001 | 18.7% | 70,556 | 15,683 | 22.2% | 1.19 | 1.06 | 1.33 | 1.11 | 1.03 | 1.21 |
| **Age category (Years)** |  |  |  |  |  |  |  |  |  |  |  |  |
| <15 | 428 | 46 | 10.7% | 2,570 | 271 | 10.5% | 0.98 | 0.70 | 1.37 | 0.98 | 0.72 | 1.33 |
| 15-29 | 2,864 | 614 | 21.4% | 17,729 | 4,685 | 26.4% | 1.23 | 1.05 | 1.44 | 1.14 | 1.00 | 1.30 |
| 30-44 | 3,274 | 641 | 19.6% | 20,373 | 4,797 | 23.5% | 1.20 | 1.09 | 1.33 | 1.11 | 1.03 | 1.20 |
| 45-59 | 2,333 | 450 | 19.3% | 16,821 | 3,641 | 21.6% | 1.12 | 0.99 | 1.27 | 1.07 | 0.95 | 1.20 |
| 60-69 | 1,574 | 234 | 14.9% | 11,609 | 2,121 | 18.3% | 1.23 | 1.06 | 1.43 | 1.16 | 1.02 | 1.33 |
| >75 | 202 | 16 | 7.9% | 1,454 | 168 | 11.6% | 1.46 | 0.74 | 2.88 | 1.32 | 0.67 | 2.61 |
| **Gender** |  |  |  |  |  |  |  |  |  |  |  |  |
| Female | 3,842 | 567 | 14.8% | 56,584 | 12,490 | 22.1% | 1.16 | 0.98 | 1.38 | 1.11 | 0.96 | 1.28 |
| Male | 6,833 | 1,434 | 21.0% | 13,972 | 3,193 | 22.9% | 1.19 | 1.08 | 1.31 | 1.12 | 1.04 | 1.20 |
| **Past history of anti TB treatment** |  |  |  |  |  |  |  |  |  |  |  |  |
| No | 9,951 | 1,743 | 17.5% | 58,634 | 11,609 | 19.8% | 1.13 | 1.00 | 1.28 | 1.14 | 1.01 | 1.28 |
| Yes | 724 | 258 | 35.6% | 11,922 | 4,074 | 34.2% | 0.96 | 0.69 | 1.33 | 0.98 | 0.72 | 1.34 |
| **Type of referring provider** |  |  |  |  |  |  |  |  |  |  |  |  |
| Public | 8,926 | 1,675 | 18.8% | 56,584 | 12,490 | 22.1% | 1.18 | 1.05 | 1.32 | 1.11 | 1.00 | 1.23 |
| Other | 1,749 | 326 | 18.6% | 13,972 | 3,193 | 22.9% | 1.23 | 0.99 | 1.53 | 1.14 | 0.95 | 1.37 |
| **Geographical distribution** |  |  |  |  |  |  |  |  |  |  |  |  |
| Urban | 3,609 | 693 | 19.2% | 28,761 | 6,643 | 23.1% | 1.20 | 1.02 | 1.41 | 1.07 | 0.97 | 1.17 |
| Rural | 4,165 | 764 | 18.3% | 27,322 | 5,803 | 21.20% | 1.16 | 0.95 | 1.41 | 1.13 | 0.96 | 1.32 |
| Tribal/Hilly | 2,901 | 544 | 18.8% | 14,473 | 3,237 | 22.40% | 1.19 | 0.98 | 1.45 | 1.12 | 0.93 | 1.34 |

n=81,231; 562 patients with missing values for history of anti-TB treatment were excluded

# Adjusted for clustering at TU level, AND adjusted for age, sex and past TB history.

Abbreviations: Suspects= number of presumptive pulmonary TB patients tested; All PTB= All diagnosed cases of pulmonary tuberculosis; Bact+PTB=bacteriologically confirmed pulmonary tuberculosis; RR=adjusted relative risk ratio; 95% CI = 95% confidence interval

**Table B: Number of presumptive tuberculosis and presumptive drug-resistant tuberculosis patients tested, population-time at risk, and tuberculosis patients with a positive Xpert MTB/RIF positive signal for rifampicin resistance diagnosed during the baseline and intervention periods at each Treatment Unit, and the incidence risk ratios comparing the intervention period to the baseline period**

|  | BASELINE PERIOD |  |  |  | INTERVENTION PERIOD |  |  |  |  |
| --- | --- | --- | --- | --- | --- | --- | --- | --- | --- |
| TU | Number of presumptive TB and DR patients tested | Person-years (Total TU population)* | Number of cases of Rif resistant PTB diagnosed | Incidence risk of diagnosed Rif resistant TB per 100,000 population | Number of presumptive TB and DR patients tested | Person-years (Total TU population)* | Number of cases of Rif resistant PTB diagnosed§ | Incidence risk of diagnosed Rif resistant TB per 100,000 population§ | IRR -Rif res TB§† |
| Urban |  |  |  |  |  |  |  |  |  |
| 1 | 1030 | 121746 | 5 | 4.1 | 7441 | 887712 | 112 | 12.6 | 3.1 |
| 2 | 1059 | 149529 | 4 | 2.7 | 10307 | 1069816 | 101 | 9.5 | 3.5 |
| 3 | 508 | 90525 | 5 | 5.5 | 2804 | 642169 | 112 | 17.4 | 3.1 |
| 4 | 1121 | 137344 | 7 | 5.1 | 9153 | 1284902 | 324 | 25.3 | 5.0 |
| Rural |  |  |  |  |  |  |  |  |  |
| 5 | 534 | 187540 | 0 | 0.0 | 2836 | 604618 | 48 | 7.9 |  |
| 6 | 776 | 119713 | 2 | 1.7 | 6822 | 880634 | 28 | 3.2 | 1.9 |
| 7 | 1343 | 129361 | 5 | 3.9 | 10443 | 979011 | 99 | 10.1 | 2.6 |
| 8 | 433 | 124201 | 0 | 0.0 | 1572 | 483836 | 16 | 3.3 |  |
| 9 | 596 | 208846 | 1 | 0.5 | 3053 | 1161870 | 23 | 2.0 | 4.1 |
| 10 | 583 | 86060 | 1 | 1.2 | 2906 | 741209 | 76 | 10.3 | 8.9 |
| Tribal/Hilly |  |  |  |  |  |  |  |  |  |
| 11 | 1002 | 133933 | 0 | 0.0 | 3894 | 508877 | 15 | 2.9 |  |
| 12 | 461 | 49381 | 0 | 0.0 | 1996 | 285579 | 31 | 11.0 |  |
| 13 | 335 | 113172 | 0 | 0.0 | 2705 | 736074 | 10 | 1.4 |  |
| 14 | 1126 | 116323 | 2 | 1.7 | 6022 | 756570 | 168 | 22.2 | 12.9 |
| Total^ | 10907 | 1767676 | 32 | 1.9 | 71954 | 11022876 | 1164 | 9.9 |  |

* Number of person-years is calculated from TU population size multiplied by time in baseline or intervention period. The population size is adjusted for 1.27% annual population growth during the study period (adjusted on a monthly basis, assuming linear increase)

§ Adjustment for difference in the distribution of characteristics of presumptive TB patients between intervention and baseline by inverse probability weighting (standardization of the intervention patient populaton to the baseline patient population)

^ IR calculated as cluster-averaged mean

bact+=bacteriologically confirmed; RIF=rifampicin; IR=incidence risk; IRR=incidence rate ratio; PTB=pulmonary tuberculosis

† An overall adjusted IRR could not be calculated due to sites with 0 DR cases during the baseline phase

**Figure A – RNTCP Diagnostic Algorithm**


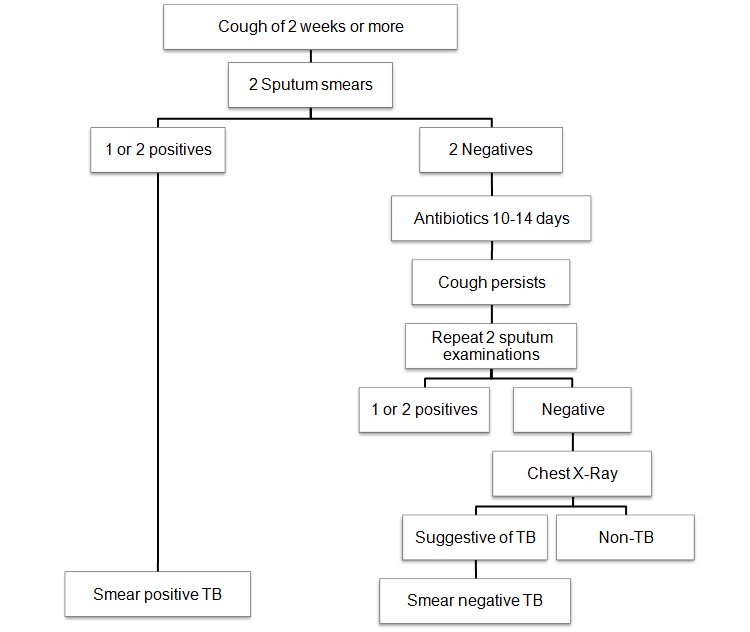


**Figure B – Study Diagnostic Algorithm**


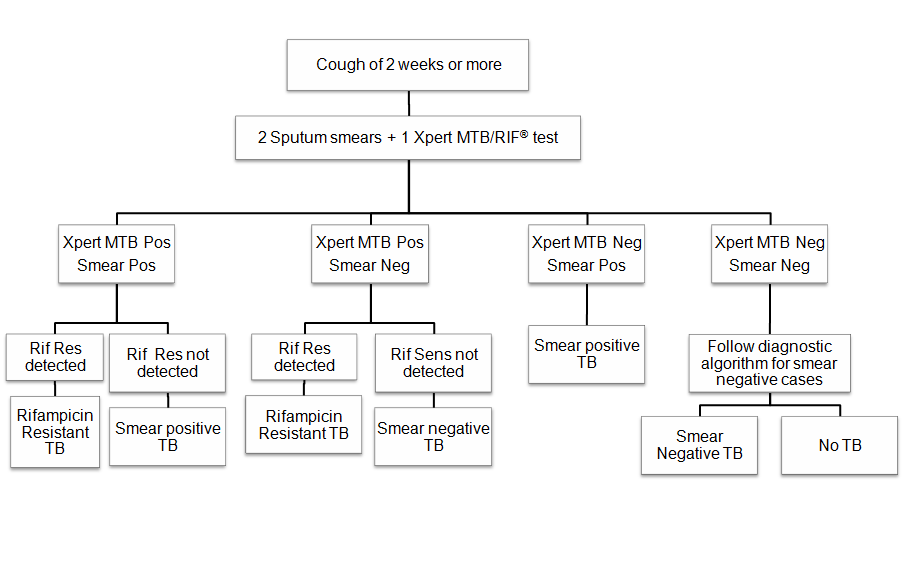


**Figure C: Transition of sites from baseline to intervention phase of the study**


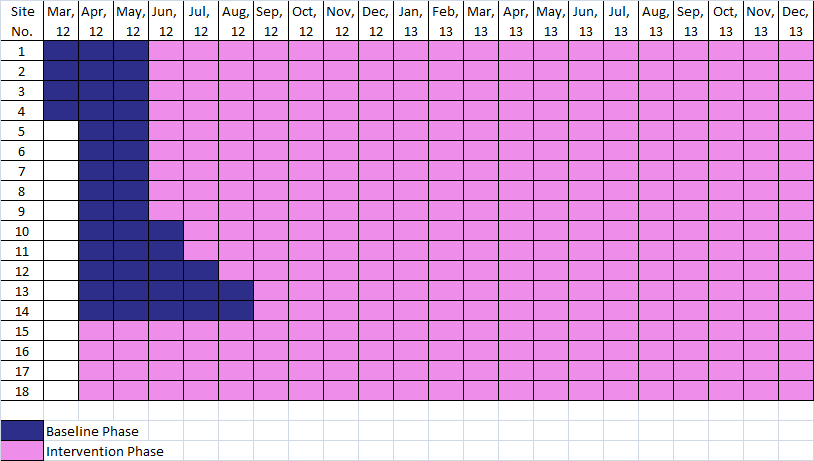

Supplement: S1 File — Annexure A, Sensitivity Analysis. Table A, Proportion of all pulmonary tuberculosis cases diagnosed at the 14 study treatment units with data collection both in the baseline and intervention phase. Table B, Number of presumptive tuberculosis and presumptive drug-resistant tuberculosis patients tested, population-time at risk, and tuberculosis patients with a positive Xpert MTB/RIF positive signal for rifampicin resistance diagnosed during the baseline and intervention periods at each Treatment Unit, and the incidence risk ratios comparing the intervention period to the baseline period. Fig A, RNTCP diagnostic Algorithm. Fig B, Study diagnostic Algorithm. Fig C, Transition of sites from baseline to intervention phase of the study. (DOCX) [file pone.0126065.s001.docx]
